# Cost-effectiveness of utidelone and capecitabine versus monotherapy in anthracycline- and taxane-refractory metastatic breast cancer

**DOI:** 10.3389/fphar.2024.1303808

**Published:** 2024-07-11

**Authors:** Mulan Chen, Heng Zhang, Xiaoyan He, Yingtao Lin

**Affiliations:** ^1^ Department of Medical Oncology, Clinical Oncology School of Fujian Medical University, Fujian Cancer Hospital, Fuzhou, China; ^2^ Clinical Medical Research Center, Clinical Oncology School of Fujian Medical University, Fujian Cancer Hospital, Fuzhou, China; ^3^ Department of Endocrinology, Fuqing City Hospital of Fujian, Fuqing City Hospital Affiliated to Fujian Medical University, Fuqing, China

**Keywords:** breast cancer, cost-effectiveness, partitioned survival model, utidelone, capecitabine

## Abstract

**Background:**

This study aimed to assess the cost-effectiveness of combining utidelone with capecitabine, compared to capecitabine monotherapy, for the treatment of anthracycline- and taxane-refractory metastatic breast cancer within the Chinese healthcare system.

**Methods:**

A partitioned survival model was formulated based on patient characteristics from the NCT02253459 trial. Efficacy, safety, and health economics data were sourced from the trial and real-world clinical practices. We derived estimates for costs, quality-adjusted life years (QALYs), and the incremental cost-effectiveness ratio (ICER) for the two treatment strategies. Sensitivity and subgroup analyses were conducted to rigorously evaluate uncertainties' impact.

**Results:**

Over a 5-year span, the combination therapy manifested substantially higher costs than capecitabine monotherapy, with a differential of US$ 26,370.63. This combined approach conferred an additional 0.49 QALYs, resulting in an ICER of US$ 53,874.17/QALY. Utilizing the established willingness-to-pay threshold, the combination might not consistently be deemed cost-effective when juxtaposed against monotherapy. However, at an ICER of US$ 53,874.4/QALY, the probability of the combination being cost-effective increased to 48.97%. Subgroup analysis revealed that the combination was more cost-effective than capecitabine alone in specific patient groups, including those <60 years, patients with more than two chemotherapy rounds, patients lacking certain metastases, patients having limited metastatic sites, patients with an Eastern Cooperative Oncology Group status of 0, and patients with particular hormone receptor profiles.

**Conclusion:**

Although the combination of utidelone and capecitabine may not be an economically viable universal choice for anthracycline- and taxane-refractory metastatic breast cancer, it could be more cost-effective in specific patient subgroups than capecitabine monotherapy.

## 1 Introduction

Breast cancer is the predominant malignant tumor in women globally and ranks among the principal causes of female mortality (Global Burden of Disease Cancer et al., 2018; Xia et al., 2022). An estimated 30%–40% of the patients diagnosed with early-stage breast cancer in China evolve to the metastatic phase. The median post-diagnosis survival duration for these individuals is approximately 2–3 years (Sung et al., 2021). Thus, extending survival and elevating the quality of life are core therapeutic targets. Despite the advancements in targeted and immunotherapies, chemotherapy remains integral to the management of advanced breast cancer (De Abreu et al., 2013; Tosello et al., 2018).

Most of these patients undergo treatment with anthracyclines and taxanes in earlier disease phases. However, challenges such as drug resistance and cumulative toxicities persistently shape treatment strategies (Roche and Vahdat, 2011; Twelves et al., 2016). Utidelone is a contemporary epothilone class microtubule inhibitor, marking its stance as a potent, broad-spectrum, anti-neoplastic agent. This class I drug, indigenously crafted in China, showcases an anti-tumor mechanism analogous to that of paclitaxel. However, its superiority is evident, especially against tumors resistant to paclitaxel and a spectrum of other chemotherapeutic agents. Preliminary studies and clinical trials up to Phase II have inferred that utidelone holds promise for those with refractory breast cancer after multiple treatment failures. Subsequently, a pivotal Phase III trial, orchestrated by Professor Xu Binhe’s team, emphasized the enhanced efficacy of a utidelone-capecitabine combination for refractory metastatic breast cancer (Zhang et al., 2017). The results of the NCT02253459 trial showed a significant improvement in progression-free survival (PFS) and overall survival (OS) for the combination therapy group. These efficacy outcomes form the basis of our cost-effectiveness analysis.

Given the profound results, the National Medical Products Administration endorsed the use of utidelone, in combination with capecitabine, for anthracycline or taxane pre-treated advanced breast cancer patients, as of 12 March 2021. This endorsement steered its incorporation into guidelines by esteemed bodies such as the Chinese Society of Clinical Oncology and the China Anti-Cancer Association. With utidelone’s price revision in 2023 and its incorporation into health insurance policies, along with the maturation of prior clinical study data, this therapeutic combination has witnessed heightened recommendation. Consequently, its clinical utilization has amplified, revolutionizing the therapeutic paradigm for advanced human epidermal growth factor receptor 2 (HER2)-negative breast cancer in China. Nevertheless, superior treatments often bear significant economic burdens (Zhang et al., 2021). In an era of escalating healthcare expenses, the financial implications on patients remain a salient concern. Although clinical benefits are of utmost significance, economic ramifications demand equal scrutiny, particularly for policymakers striving for a balance between therapeutic quality and expenditure. Exorbitant treatments, irrespective of their clinical prowess, may face hesitancy in adoption. Contemplating these dynamics and anchoring on the NCT02253459 dataset (Zhang et al., 2017), our research endeavors to discern the cost-efficacy of the utidelone-capecitabine duo vis-à-vis capecitabine monotherapy, encompassing clinical and economic dimensions within the Chinese healthcare milieu.

Our study was conducted at Fujian Cancer Hospital, Fuzhou, China. The study was designed by referring to the International Council for Harmonization E6 guidelines for Good Clinical Practice, the Declaration of Helsinki principles, and applicable laws and regulations (World Medical, 2013). The reporting criteria of the Consolidated Health Economic Evaluation Reporting Standards were followed when writing the economic evaluation section (Husereau et al., 2013).

## 2 Materials and methods

### 2.1 Target population

The target population for our study was modeled after the NCT02253459 clinical trial. The NCT02253459 trial was a Phase III, multicenter, open-label, randomized controlled study designed to demonstrate superiority. It encompassed 26 hospitals across China, with Fujian Cancer Hospital being one of the participating centers. Participants eligible for the study were females, aged 18–70 years, who had a confirmed histological or cytological diagnosis of metastatic breast cancer. Before their inclusion, these participants must have undergone up to four different chemotherapy regimens, including treatments with both anthracycline and taxane. For the purposes of this stipulation, both adjuvant and neoadjuvant treatments were considered a single therapeutic regimen. Additional eligibility criteria included an Eastern Cooperative Oncology Group (ECOG) performance status ranging from 0 to 2, a projected life expectancy of at least 3 months, a minimum of one lesion that could be assessed through imaging, and a peripheral neuropathy grade below grade 2 as per the Common Terminology Criteria for Adverse Events version 4.03. All these criteria were evaluated within the 4 weeks before randomization. Individuals were excluded if they had received any form of chemotherapy, radiotherapy, hormone therapy, or targeted molecular therapy in the preceding 4 weeks. Those who did not respond favorably to standard capecitabine treatment or had ceased an effective standard capecitabine regimen less than 6 months before were also excluded. Participants with significant cardiac, pulmonary, hepatic, or renal complications; unmanaged hypertension or diabetes; severe gastrointestinal ulcers; ongoing infections requiring antibiotic treatment; uncontrollable cerebral or bone metastases; significant psychiatric conditions; or those who were either pregnant or lactating were deemed ineligible.

### 2.2 Intervention

Participants were randomized in a 2:1 ratio to either the utidelone plus capecitabine group or the capecitabine monotherapy group. Eligible patients underwent central, sequential randomization with stringent protocols; this randomization was constrained to block sizes of six. Although the patients were not stratified, pre-defined subgroups were assessed at the study’s conclusion. Treatment for both study groups was administered in 21-day cycles. The combination therapy group was administered utidelone at a dosage of 30 mg/m^2^ intravenously once daily from days 1–5, supplemented with capecitabine at 1,000 mg/m^2^ orally twice daily from days 1–14. The monotherapy group was administered capecitabine at 1,250 mg/m^2^ orally twice daily from days 1–14. Patients persisted with the designated study treatment until there was evidence of disease progression, onset of intolerable toxicity, or if cessation was requested by the patient or the overseeing investigator.

### 2.3 Model construction

**FIGURE 1:**
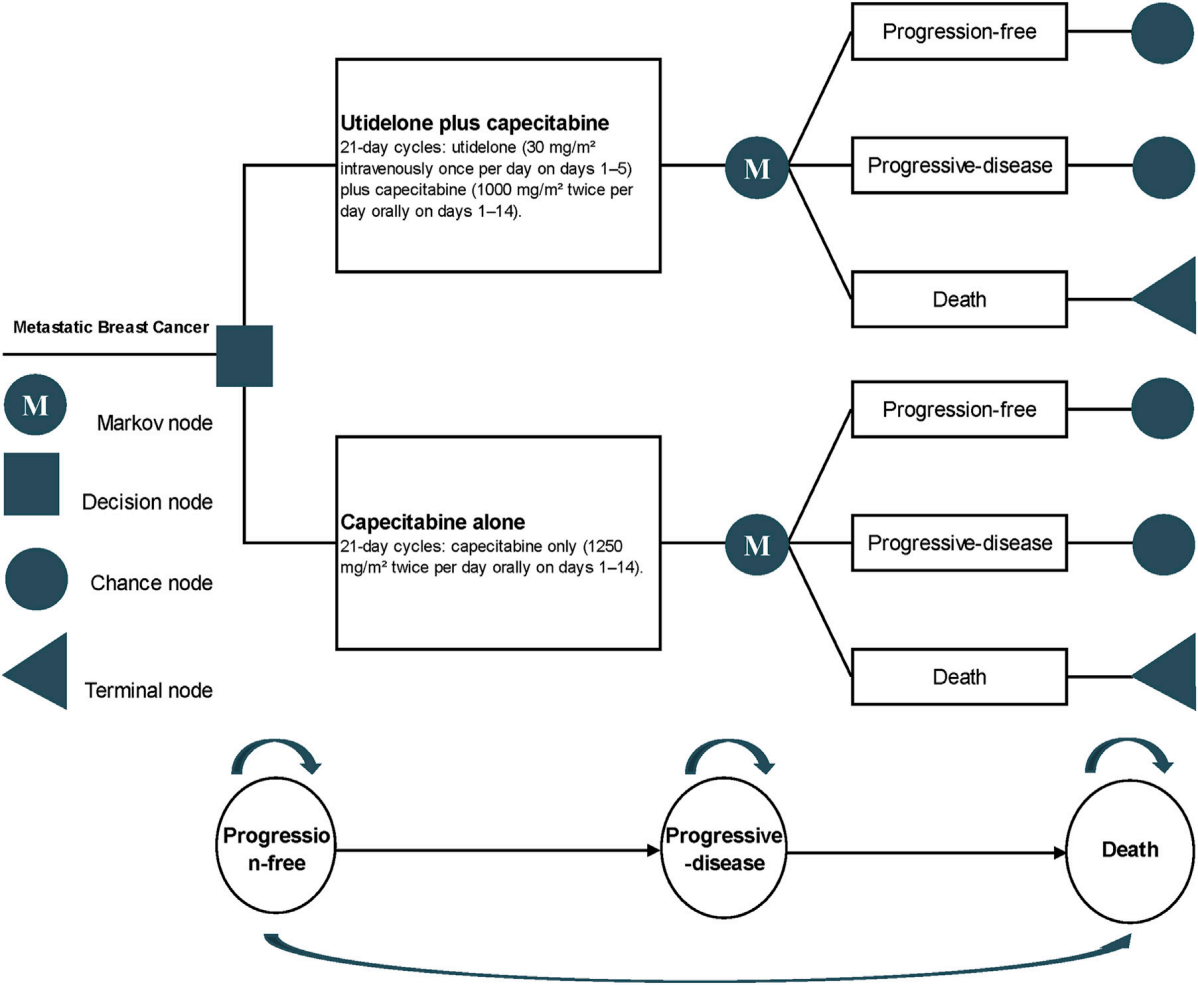
Profile of the partitioned survival model for NCT02253459 trial.

The cost-effectiveness was evaluated using a partitioned survival model derived from the NCT02253459 trial data (Partitioned survival model, 2016). This model is routinely employed to assess the financial and efficacy outcomes in metastatic oncology research (Insinga et al., 2018; Chouaid et al., 2019; Loong et al., 2020; Cai C. et al., 2021). It delineates three distinct health states (Figure 1): the progression-free state (from patient entry until the onset of disease progression), the progressive disease (PD) state (spanning the time the patient remains alive post the initiation of disease progression), and the terminal state. Each cycle within the model is pegged at 21 days, with a time horizon set at 5 years, mirroring the timeline of the NCT02253459 trial. Principal outputs from the model include cost, quality-adjusted life years (QALYs), and the incremental cost-effectiveness ratio (ICER).

### 2.4 Cost assessment

Our evaluation integrated an assortment of clinical expenses associated with cancer treatment. These costs encompassed drug acquisition, laboratory tests, radiological evaluations, medication administration, consultations related to disease progression, treatment-related adverse events (AEs), and end-of-life care expenses (terminal cost). We designated these as direct medical expenditures, converting them to US dollars using the August 2023 exchange rate: 1 USD = 7.238 RMB. Our financial data was sourced from esteemed entities, such as the National Health Commission of China, the Health Commission of Fujian Province, and expert consensus. Detailed cost parameters are presented in Table 1.

**TABLE 1:** Primary input parameters for our model and ranges for sensitivity analysis.

Input parameters	Base case value	Lower bound	Upper bound	Distribution	Source
Log-Logistic OS survival model					
Utidelone plus capecitabine	Scale (λ) = 15.224; Shape (γ) = 2.826	-	-	Log-Logistic	Zhang et al. (2017)
Capecitabine alone	Scale (λ) = 12.534; Shape (γ) = 2.933	-	-	Log-Logistic	Zhang et al. (2017)
Weibull PFS survival model					
Utidelone plus capecitabine	Scale (λ) = 0.0345; Shape (γ) = 1.51337	-	-	Weibull	Zhang et al. (2017)
Capecitabine alone	Scale (λ) = 0.072; Shape (γ) = 1.3696	-	-	Weibull	Zhang et al. (2017)
Drug acquisition, US$					
Utidelone (Chengdu Biostar Technologies, Ltd.) per 50 mg	408.94	327.15	490.72	Gamma	National Health Commission of China
Capecitabine (Roche Pharmaceuticals Co., Ltd.) per 500 mg	36.51	29.21	43.81	Gamma	National Health Commission of China
Preventive medication, US$					
Cimetidine (Shijiazhuang Kangli Pharmaceutical Co., Ltd.) per 100 mg	3.38	2.70	4.05	Gamma	National Health Commission of China
Diphenhydramine (Hebei Meitu Pharmaceutical Co., Ltd.) per 20 mg	4.69	3.76	5.63	Gamma	National Health Commission of China
Dexamethasone (Anhui Yangtze River Pharmaceutical Co., Ltd.) per 5 mg	0.99	0.79	1.19	Gamma	National Health Commission of China
Drug administration, US$					
Drug administration Hospitalization	17.27	13.82	20.72	Gamma	Local medical data
Drug administration Infusion	1.64	1.31	1.96	Gamma	Local medical data
Laboratory and imaging examination, US$					
12-lead ECG	3.73	2.98	4.48	Gamma	Fujian Provincial Health Commission
Hematology	3.45	2.76	4.14	Gamma	Fujian Provincial Health Commission
Serum chemistry	24.87	19.89	29.84	Gamma	Fujian Provincial Health Commission
Urinalysis	4.14	3.32	4.97	Gamma	Fujian Provincial Health Commission
Contrast-enhanced CT	296.96	237.56	356.35	Gamma	Fujian Provincial Health Commission
Costs of AE (Grade ⩾3), US$					
Anemia	275.30	220.24	330.36	Gamma	Xu et al. (2020), Gradishar et al. (2022)
Neutropenia	483.54	386.83	580.25	Gamma	Xu et al. (2020), Gradishar et al. (2022)
Diarrhoea	10.36	8.29	12.43	Gamma	Xu et al. (2020), Gradishar et al. (2022)
Peripheral neuropathy	621.69	497.35	746.03	Gamma	Xu et al. (2020), Gradishar et al. (2022)
Palmar-plantar erythrodysesthesia	93.25	74.60	111.90	Gamma	Xu et al. (2020), Gradishar et al. (2022)
Leucopenia	483.54	386.83	580.25	Gamma	Xu et al. (2020), Gradishar et al. (2022)
Utidelone plus capecitabine group AE risks (grade ⩾3)					
Anemia	0.034	0.027	0.04	Beta	Zhang et al. (2017)
Neutropenia	0.116	0.093	0.14	Beta	Zhang et al. (2017)
Diarrhoea	0.071	0.057	0.09	Beta	Zhang et al. (2017)
Peripheral neuropathy	0.217	0.174	0.26	Beta	Zhang et al. (2017)
Palmar-plantar erythrodysesthesia	0.067	0.054	0.08	Beta	Zhang et al. (2017)
Leucopenia	0.052	0.042	0.06	Beta	Zhang et al. (2017)
Capecitabine alone group AE risks (grade ⩾3)					
Anemia	0.031	0.025	0.04	Beta	Zhang et al. (2017)
Neutropenia	0.092	0.074	0.11	Beta	Zhang et al. (2017)
Diarrhoea	0.023	0.018	0.03	Beta	Zhang et al. (2017)
Peripheral neuropathy	0.008	0.006	0.01	Beta	Zhang et al. (2017)
Palmar-plantar erythrodysesthesia	0.077	0.062	0.09	Beta	Zhang et al. (2017)
Leucopenia	0.054	0.043	0.06	Beta	Zhang et al. (2017)
Terminal cost, US$					
End-of-life care	1,036.15	828.92	1,243.39	Gamma	Lang et al. (2023)
Utility value					
Progression-free disease	0.85	0.68	1.02	Beta	Li et al. (2019)
Progressive disease	0.69	0.55	0.83	Beta	Liu et al. (2017)
Disutility due to Grade ⩾3 AEs	−0.28	−0.22	−0.34	Beta	Wu and Ma (2020)
Discount rate	0.05	0	0.08	Beta	Liu et al. (2020)

Abbreviations: OS, overall survival; PFS, progression-free survival; ECG, electrocardiogram; CT, computed tomography; AE, adverse events.

For our analysis, Chengdu Biostar Technologies, Ltd. was the manufacturer of utidelone, whereas Roche Pharmaceuticals Co., Ltd. produced capecitabine. We referenced the 2023 Drug Price Directory from the National Health Commission for drug pricing, highlighting utidelone at US$ 409/50 mg and capecitabine at US$ 37/500 mg. The dosage and strength were predicated on the findings of the NCT02253459 trial. Since the aforementioned trial did not specify body surface area and weight, we estimated the body surface area to be 1.73 m^2^ based on average Chinese demographics from the “China Statistical Yearbook 2022” published by the National Bureau of Statistics of China.

Our model simulates real-world drug administration logistics, encapsulating costs for hospitalization, nursing care, and drug infusion. The unique formulation of utidelone, which contains the Cremophor EL polyoxyethylated castor oil allergen, necessitates pre-treatment precautions. Consequently, to mitigate potential allergic reactions, patients received a pre-medication regimen before each utidelone administration. Drug dosages were adjusted on subsequent days based on individual patient reactions. Furthermore, drug wastage was factored in by rounding off drug quantities to the nearest vial size, accounting for the standard procedure of discarding surplus drugs post-infusion.

Based on the NCT02253459 trial protocol, we presumed a consistent schedule for laboratory and imaging examinations. In each treatment cycle, patients underwent an electrocardiogram (ECG), hematology tests, serum chemistry assessments, and urinalysis. The methodology for imaging remained uniform throughout our study. Specifically, contrast-enhanced computed tomography (CT) scans—encompassing the neck, chest, and abdomen—were performed once every two treatment cycles and continued until either disease progression or the patient’s demise.

AE-associated treatment costs were calculated leveraging the 2023 charging standards set by the Fujian Provincial Health Commission. We took into account treatment-related AEs (grade 3 or higher) with an incidence rate surpassing 5% while also incorporating the costs of other prevalent AEs. We based our assumption that AEs primarily manifested in the initial treatment cycle (Durkee et al., 2016; Mistry et al., 2018). The list of AEs included anemia, neutropenia, and peripheral neuropathy, among others. Costs specific to treatment-induced AEs were drawn from expert consensus.

Our projection anticipated costs pertaining to medical consultations upon disease progression and end-of-life care. These terminal care expenses were gauged using the 2023 charging standards from the Fujian Provincial Health Commission.

### 2.5 Utility scores

Utility scores quantify the quality of life associated with distinct health states. Although the NCT02253459 trial did not supply specific patient utility score data, researchers have leveraged quality of life data from the literature as benchmarks for utility scores in cost-effectiveness evaluations of breast cancer treatments (Phua et al., 2020; Yang et al., 2023). In our model, these scores are sourced from established breast cancer utility evaluations. Specifically, the utility score for PFS is 0.85, that for PD is 0.69, and that for death is 0 (Liu et al., 2017; Li et al., 2019). Furthermore, we considered the detrimental effects of grade 3 or higher AEs on a patient’s quality of life. Such detrimental impacts manifest as negative utility scores (Wu and Ma, 2020). The key utility parameters can be found in Table 1.

### 2.6 Sensitivity analyses

We executed a deterministic sensitivity analysis on our model by systematically varying all input parameters within a range of ±20% (Mohan and Chattopadhyay, 2020; Cai H. et al., 2021). By isolating each parameter’s adjustment while maintaining other variables at their base values, we gauged the influence of individual parameters on model stability. The annual discount rate for both costs and health outcomes was set at 5%, with a sensitivity range extending from 0% to 8% (Liu et al., 2020). Additionally, we conducted a probabilistic sensitivity analysis using Monte Carlo simulations (Wu et al., 2022; Zheng et al., 2022). In this analysis, cost parameters were assumed to follow a gamma distribution, whereas utility parameters were modeled using a beta distribution (Briggs et al., 2012). For each simulation iteration, values for all parameters were simultaneously and randomly drawn from their respective distributions. After completing 10,000 iterations, we analyzed the combined impact of these parameter variations to assess the model’s resilience.

### 2.7 Subgroup analyses

In our subgroup analysis, the ICER was determined using the subgroup-specific hazard ratios (HRs) sourced from the NCT02253459 trial. Owing to data constraints, we incorporated methodologies from the existing literature and postulated that the HRs for PFS within the subgroups mirrored those of the overall cohort (Shao et al., 2023). Subgroups were delineated based on criteria such as age, number of prior chemotherapy treatments, presence of visceral metastasis, lymph node involvement, count of metastatic sites, ECOG performance status, history of capecitabine treatment, HER2 status, and hormone receptor presence. Owing to limited data, we operated under proportional hazard assumptions. The analysis was anchored to a scenario where the willingness-to-pay (WTP) threshold was benchmarked at thrice the mean Gross Domestic Product (GDP) of China, amounting to US$ 35,519.

### 2.8 Statistical analysis

We employed the Get Data Graph Digitizer software (version 2.26, http://getdata-graph-digitizer.software.informer.com/) to extract survival curves from the NCT02253459 trial data. Using R software (version 4.2.2, https://www.r-project.org/), individual patient data were reconstructed to model patient survival rates under various distributions: Weibull, log-Normal, log-Logistic, Gompertz, Gamma, and Exponential. The appropriate distribution was determined based on achieving the minimum values for the Akaike Information Criterion and the Bayesian Information Criterion. In addition to these statistical criteria, the selection was further guided by visual inspection and considerations from existing literature. Based on these assessments, the Weibull distribution was chosen to model a 5-year PFS for patients treated with both utidelone and capecitabine and for those receiving only capecitabine. The log-Logistic distribution was adopted to represent a 5-year OS for both patient groups. Computations of costs and health outcomes for the three distinct health states and results from the subgroup and sensitivity analyses were performed using Excel (version 2019).

## 3 Results

### 3.1 Base-case analysis

In the NCT02253459 study, the median PFS for the combination of utidelone and capecitabine was 7.72 months (95% CI: 6.47–8.11) versus 4.76 months (95% CI: 4.07–6.08) for the capecitabine monotherapy group. The median OS reached 16.13 months (95% CI: 13.54–17.05) in the combination group, in contrast to 12.78 months (95% CI: 11.14–14.32) in the monotherapy group up to the point of data cut-off. Our model’s simulation of a hypothetical cohort showed survival results closely aligned with the actual clinical trial data: 7.7 months median PFS in the combination group and 4.9 months in the monotherapy group. The median OS was 15.4 and 12.6 months for the combination and monotherapy groups, respectively.

Over a 5-year period, the combined utidelone and capecitabine treatment incurred costs of US$ 33,216.41, whereas the capecitabine monotherapy amounted to US$ 6,845.78, resulting in an incremental cost of US$ 26,370.63. The cost breakdown between the combination therapy and monotherapy, respectively, was as follows: drug acquisition (US$ 27,177.38 vs. US$ 3,327.61), drug administration (US$ 1,556.73 vs. US$ 0), laboratory tests and radiological evaluations (US$ 2,142.27 vs. US$ 1,603.13), treatment-related AEs (US$ 855.63 vs. US$ 477.81), PD management (US$ 496.50 vs. US$ 439.50), and terminal care (US$ 987.90 vs. US$ 997.74). The combination treatment resulted in a QALY increase of 0.49 (1.54 vs. 1.05) compared with monotherapy. The ICER for the combination therapy versus monotherapy was estimated at US$ 53,874.17/QALY (Table 2).

**TABLE 2:** Results of the base-case analysis.

Results	Utidelone plus capecitabine	Capecitabine alone
Total costs, US$	33,216.41	6,845.78
QALYs	1.54	1.05
ICER, US$/QALY	53,874.17	—

Abbreviations: QALY, quality-adjusted life year; ICER, incremental cost-effectiveness ratio.

### 3.2 Sensitivity analyses

#### 3.2.1 Deterministic sensitivity analyses

**FIGURE 2:**
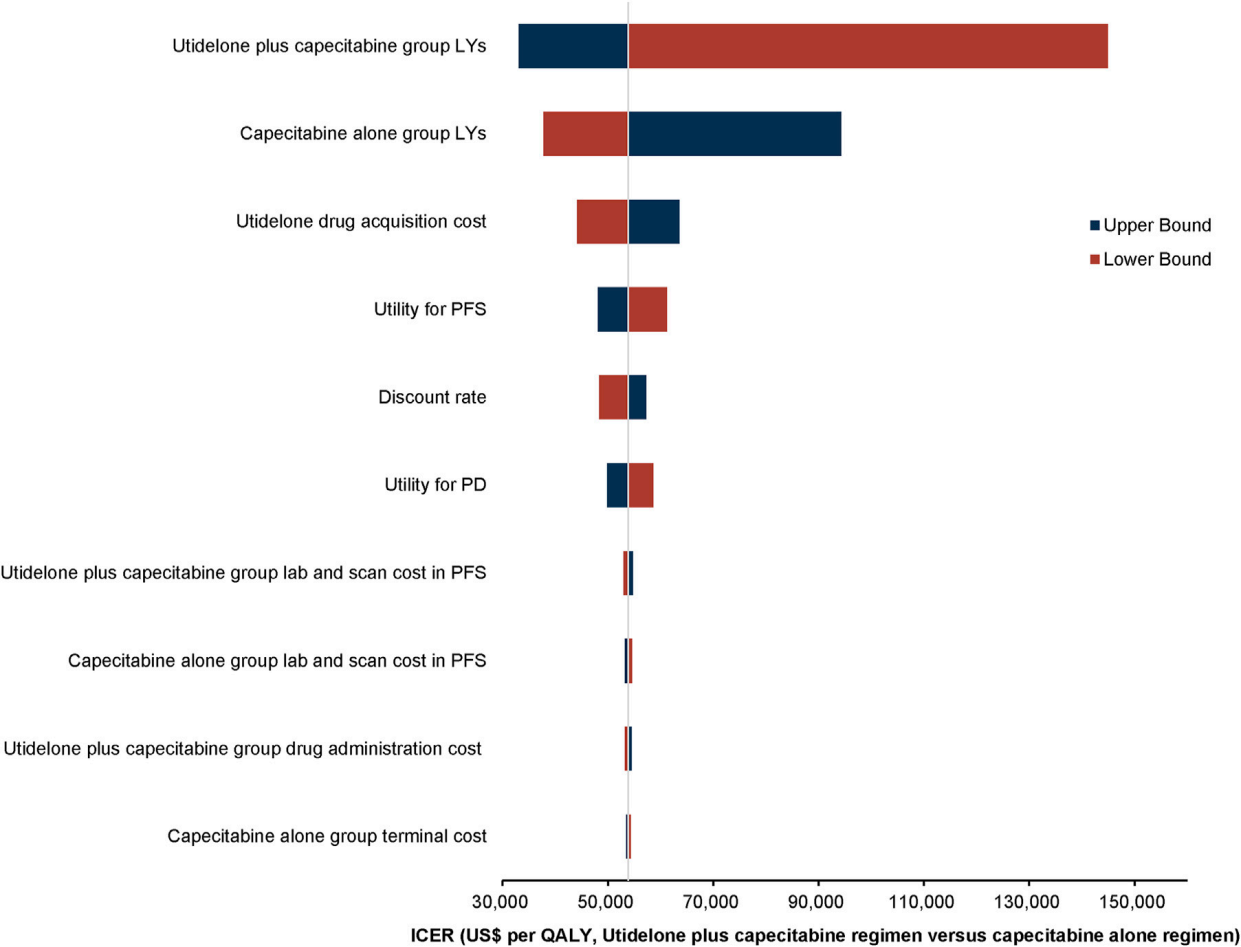
Tornado diagram depicting the top 10 most influential parameters.

One-way deterministic sensitivity analyses demonstrated that the model’s sensitivity was highest in relation to the survival duration of the utidelone plus capecitabine group. Furthermore, the analysis showed that the survival duration of the capecitabine alone group, the cost associated with utidelone drug acquisition, and the utility for PFS exerted a notable influence on the model’s outcomes. The ten parameters with the most pronounced impact are depicted in the tornado diagram (Figure 2). Adjustments to individual parameters in the model caused the ICER value to vary between US$ 33,000 and US$ 150,000.

#### 3.2.2 Probabilistic sensitivity analyses

**FIGURE 3:**
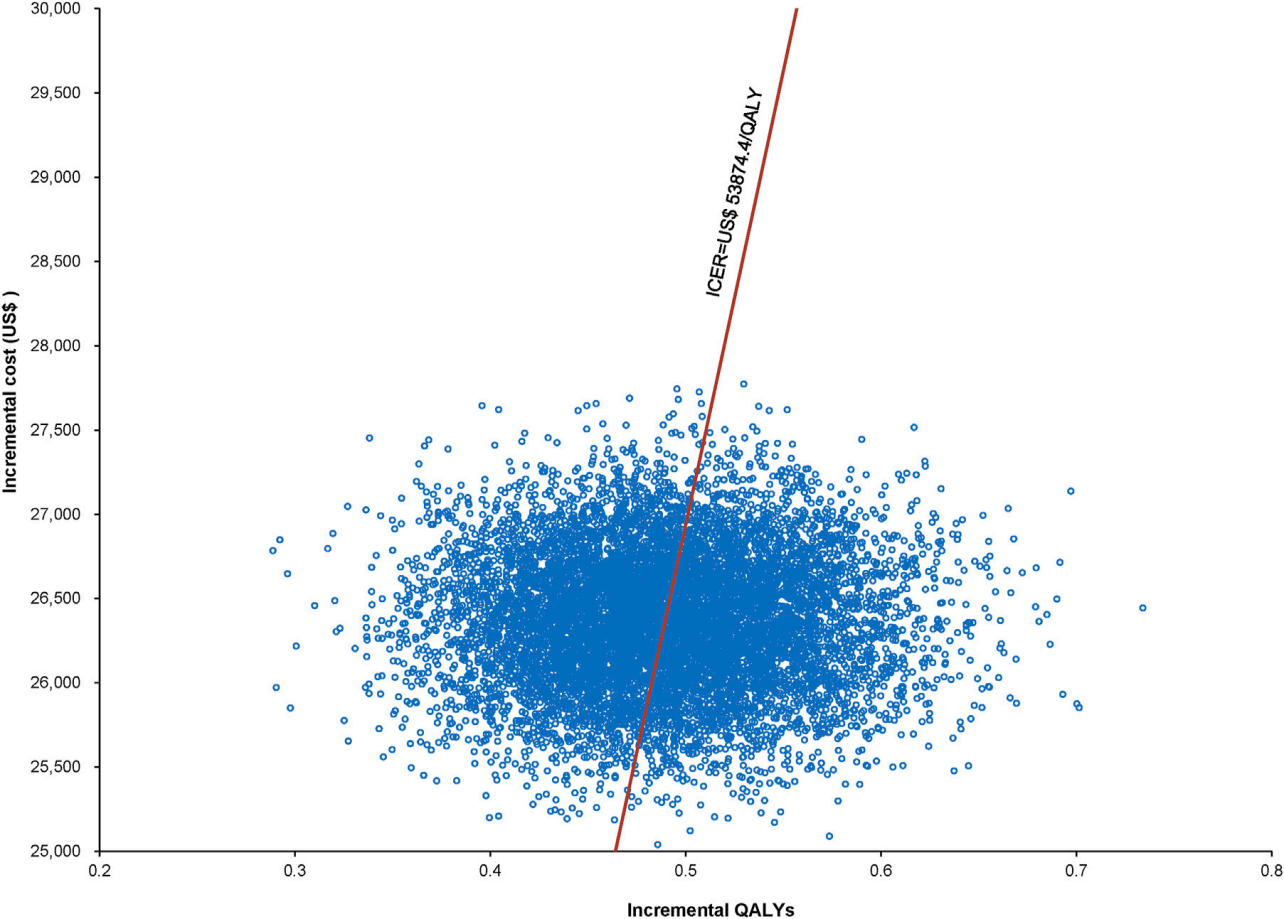
Scatter plot representing Monte Carlo sensitivity analysis.

**FIGURE 4:**
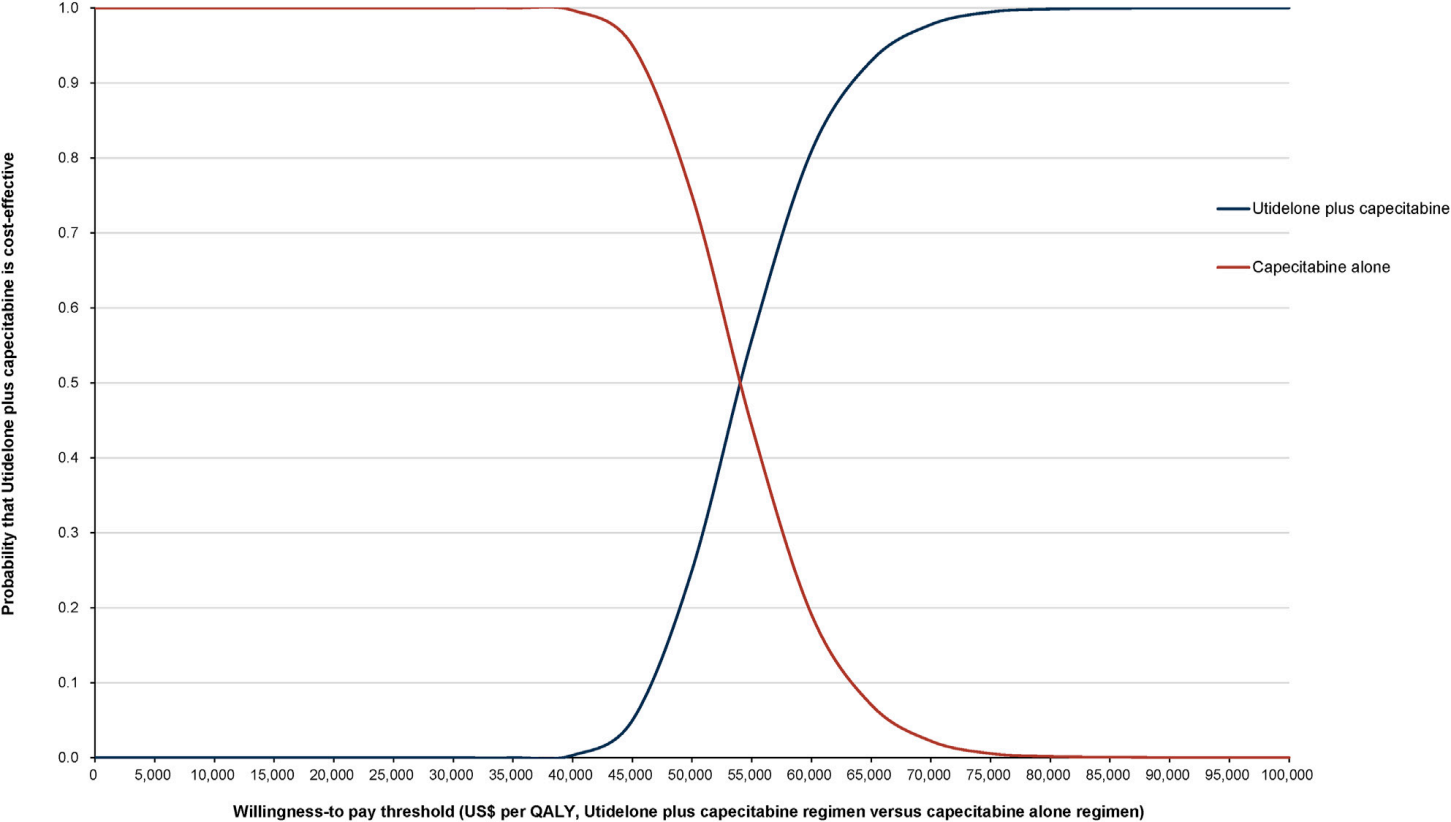
Cost-effectiveness acceptability curve comparing utidelone plus capecitabine versus capecitabine alone.

The World Health Organization (WHO) recommends a WTP threshold set at three times the GDP *per capita* (Murray et al., 2000). As of 2022, the GDP *per capita* for China was US$ 11,839.80, thereby establishing the WTP threshold at US$ 35,519.39/QALY. Insights from Monte Carlo probabilistic sensitivity analyses suggest that at this WTP threshold of US$ 35,519.39/QALY, the combination of utidelone and capecitabine may not be a cost-effective alternative to using capecitabine alone. However, adjusting the WTP threshold to US$ 53,874.4/QALY (approximating the simulated value of US$ 53,874.17/QALY), the likelihood of the utidelone and capecitabine combination being cost-effective relative to capecitabine alone rose to 48.97% (Figures 3, 4).

### 3.3 Subgroup analysis

In the subgroup analysis, based on a WTP threshold equivalent to three times China’s GDP *per capita*, the combined therapy of utidelone and capecitabine appears to be more cost-effective than capecitabine monotherapy for the following patient cohorts: those aged <60 (ICER = US$ 34,050.19/QALY), those who have undergone over two prior chemotherapy regimens (ICER = US$ 27,404.34/QALY), those without visceral or lymph node metastases (ICERs of US$ 25,188.24/QALY and US$ 31,178.75/QALY, respectively), those with two or fewer metastatic sites (ICER = US$ 34,050.19/QALY), those with an ECOG status of 0 (ICER = US$ 23,176.78/QALY), and those with HER2 and hormone receptor statuses of HER2- oestrogen receptor (OR)+ progesterone receptor (PR)+ or solely HER2- (ICERs of US$ 13,157.41/QALY and US$ 19,672.60/QALY, respectively). Conversely, the combined regimen may not present a cost-effective advantage over capecitabine monotherapy for the subsequent categories: age ≥60 (ICER = US$ 42,848.40/QALY), ≤2 previous chemotherapy regimens (ICER = US$ 42,848.40/QALY), presence of visceral metastases (ICER = US$ 40,870.92/QALY), presence of lymph node metastases (ICER = US$ 40,870.92/QALY), >2 metastatic sites (ICER = US$ 40,870.92/QALY), ECOG status ≥1 (ICER = US$ 57,939.89/QALY), both previous capecitabine treatment categories (ICERs of US$ 37,260.93/QALY and US$ 39,011.73/QALY). and those with HER2 and hormone receptor statuses of HER2- ER- PR- or HER2+ (ICERs of US$ 61160.23/QALY and US$ 82040.14/QALY, respectively). A detailed overview is provided in Table 3.

**TABLE 3:** Subgroup analysis results.

Subgroup	HR for OS (95%CI)	ICER, US$/QALY (range)	Cost-effectiveness probability of utidelone plus capecitabine (%)
Age (years)			
≥60	0.70 (0.37–1.34)	42,848.40 (10,764.67, dominated)	4.06
<60	0.65 (0.51–0.83)	34,050.19 (18,890.30, 87,512.93)	63.07
Number of previous chemotherapy regime			
>2	0.60 (0.42–0.85)	27,404.34 (13,157.41, 100,345.13)	97.69
≤2	0.70 (0.52–0.94)	42,848.40 (19,672.60, 232,827.37)	4.27
Presence of visceral metastases			
Yes	0.69 (0.53–0.89)	40,870.92 (20,490.19, 137,448.15)	9.85
No	0.58 (0.35–0.96)	25,188.24 (9,929.99, 311,794.41)	99.69
Presence of lymph node metastases			
Yes	0.69 (0.52–0.92)	40,870.92 (19,672.60, 183,811.13)	9.54
No	0.63 (0.43–0.91)	31,178.75 (13,695.12, 165,675.29)	85.28
Number of metastatic sites			
>2	0.69 (0.50-0-95)	40,870.92 (18,141.33, 267,080.69)	10.45
≤2	0.65 (0.47–0.90)	34,050.19 (16,076.98, 150,434.47)	63.45
ECOG status			
0	0.56 (0.37-0-85)	23,176.78 (10,764.67, 100,345.13)	99.96
≥1	0.76 (0.58–1.00)	57,939.89 (251,88.24, 840,806.46)	0.00
Previous capecitabine treatment			
Yes	0.67 (0.36–1.24)	37,260.93 (10,339.34, dominated)	33.36
No	0.68 (0.53–0.87)	39,011.73 (20,490.19, 116,500.38)	19.09
HER2 and hormone receptor status			
HER2- OR + PR+	0.42 (0.27–0.64)	13,157.41 (7,158.08, 32,575.32)	100.00
HER2- OR- PR-	0.77 (0.48–1.24)	61,160.23 (16,736.29, dominated)	0.00
HER2-	0.52 (0.38–0.70	19,672.60 (11,206.70, 10,764.67)	100.00
HER2+	0.82 (0.52–1.29)	82,040.14 (19,672.60, dominated)	0.00

Abbreviations: HR, hazard ratio; OS, overall survival; CI, confidence interval; ICER, incremental cost-effectiveness ratio; QALY, quality-adjusted life year; Dominated, a regimen is an absolute disadvantaged one; ECOG, eastern cooperative oncology group; HER2, human epidermal growth factor receptor 2; OR, oestrogen receptor; PR, progesterone receptor.

## 4 Discussion

In China, patients with advanced breast cancer who have previously been treated with high doses of anthracycline antibiotics and taxanes have limited drug options beyond the approved capecitabine and gemcitabine (Binder, 2013; Cardoso et al., 2018). Medications such as gemcitabine, vinorelbine, platinum compounds, etoposide, and a limited number of fluorouracil injections are accessible; however, there is limited evidence to substantiate their significant therapeutic efficacy (Rivera and Gomez, 2010; Aapro and Finek, 2012; Giannone et al., 2018; Yuan et al., 2019; Wang et al., 2022). An escalating concern for this patient population is the increasing emergence of drug resistance (Yeo et al., 2014). In response, global research entities are proactively seeking innovative compounds. These compounds, while mirroring the mechanisms of action of existing drugs, possess distinct molecular structures, aim to counteract drug resistance challenges. Utidelone and taxol exhibit similar mechanisms of action; however, their molecular configurations are markedly different (Giannakakou et al., 2000). Preclinical investigations showed that utidelone had pronounced antitumor activity against taxane-resistant cell lines and mouse tumor xenograft models. Furthermore, Phase II clinical trials have authenticated the therapeutic efficacy and safety of co-administering utidelone with capecitabine for advanced metastatic breast cancer (Zhang et al., 2016). The ensuing Phase III clinical trial data underscored that, in contrast to using capecitabine alone, the combined regimen of utidelone and capecitabine significantly augmented patients’ PFS and OS metrics (Zhang et al., 2017). Within this trial, patients with advanced disease who had prior treatments with anthracyclines and taxanes witnessed a notable extension in OS from 15.7 to 20.9 months when treated with the combined regimen, marking a 31% reduction in mortality risk. Concurrently, the PFS span expanded from 4.11 to 8.57 months, indicating a 54% diminished risk of disease advancement. The treatment’s response rate also substantially increased from 26.7% to 49.8%.

The combined administration of utidelone and capecitabine presents a novel therapeutic approach for patients with advanced metastatic breast cancer who exhibit suboptimal responses to the prevailing standard treatments (Gradishar et al., 2022). However, within the framework of China’s healthcare insurance system, this combined treatment protocol entails substantial costs. Thus, a rigorous assessment of its cost-effectiveness is paramount when deciding on the therapeutic strategy to pursue (Li et al., 2017; Yip et al., 2019). In this context, our study juxtaposed the cost-effectiveness of the combined utidelone and capecitabine regimen against the sole use of capecitabine in treating advanced metastatic breast cancer. Drawing upon the data from the NCT02253459 trial, the synergistic intervention of utidelone and capecitabine demonstrates a superior survival rate for patients with intractable metastatic breast cancer than that achieved with capecitabine monotherapy. However, this integrative approach also leads to a marked surge in medical expenditure. For patients choosing the combined therapy, the incremental cost per QALY was US$ 53,874.17 when benchmarked against capecitabine monotherapy. Viewed through the lens of China’s healthcare insurance schema, the combined therapeutic regimen may not represent a cost-efficient alternative to monotherapy chemotherapy. Delving into the results from our probability sensitivity analysis, with a WTP threshold fixed at US$ 53,874.4/QALY, the odds of the combined approach being a cost-beneficial substitute for standalone chemotherapy dwindles to a mere 48.97%. Remarkably, when the WTP benchmark is adjusted downward to US$ 35,519/QALY, this likelihood plummets to an absolute zero.

Our research findings further elucidate that while the cost-effectiveness of combining utidelone with capecitabine compared to that capecitabine alone is not notably superior within the general patient population, subgroup analyses spotlight specific cohorts that significantly excel in economic benefits. Notably, for subgroups that include patients <60 years old, those who have undergone more than two chemotherapy sessions, individuals without visceral and lymph node metastases, patients with a maximum of two metastatic sites, those with an ECOG score of 0, and those characterized by HER2- ER + PR + or HER2- statuses, the combined treatment approach is cost-effective. In contrast, for certain other subgroups, this therapeutic strategy does not demonstrate cost advantages. Deepening our analysis by considering the baseline attributes of the clinical trial participants and the intrinsic biology of their tumors, we derive two salient conclusions. First, the economically beneficial subgroups typically comprise younger patients who are in better overall health and present with a reduced tumor load. Their profile renders them more equipped to tolerate the dual-drug regimen and adhere strictly to the treatment protocol, thereby extending their survival and ultimately securing enhanced economic outcomes (Hamer et al., 2017; Sun et al., 2017). Second, these patients typically have a history of being treated with at least two distinct therapeutic approaches and are largely characterized by drug resistance. Considering their constrained and potentially less effective future treatment prospects, the incorporation of this intensified dual-drug regimen may facilitate prolonged survival (Xu et al., 2020), resulting in more substantial economic gains.

We further ascertained that, while the model suggests that the HER2- ER- PR- molecular subtype lacks notable cost-effectiveness, such findings may not align with real-world clinical scenarios. Patients characterized by the HER2- ER- PR- molecular profile typically manifest a more aggressive disease trajectory, often leading to an unfavorable prognosis and abbreviated survival durations (Xu et al., 2015). Additionally, these individuals tend to be bereft of specific targeted and endocrine therapeutic interventions in their treatment regimen. As the disease escalates to more advanced phases, their viable treatment alternatives dwindle considerably (Zhao et al., 2021). Against this context, innovative therapeutic strategies, such as the combination of utidelone and capecitabine, which hold potential to counteract drug resistance, warrant significant clinical consideration for this demographic. Nevertheless, the representation of this subgroup seemed somewhat sparse in the Phase III clinical trial, potentially skewing the precision of the subsequent analysis. Thus, for this distinct patient subset, we urge medical practitioners and policymakers not to predicate decisions purely on cost-effectiveness, thereby eschewing the combined utidelone and capecitabine treatment approach. Instead, this cost-effectiveness evaluation should serve as a pivotal instrument in brokering drug pricing discussions with healthcare insurance providers. The advent of immunotherapies, endocrine, and targeted therapies has broadened the metastatic breast cancer treatment horizon, providing new options for patients (Barzaman et al., 2020). The combination of these innovative treatments with traditional chemotherapy, exemplified by utidelone and capecitabine, highlights the necessity for personalized, adaptable treatment plans that respond to individual patient needs. Our study’s subgroup analysis indicates that specific patient groups may achieve greater economic benefits from combination therapies, emphasizing the value of biomarker and clinical factor identification to predict treatment outcomes. The quintessential aim of our research is to foster the endorsement of therapeutic regimens that authentically serve patients’ interests, emphasizing the evaluation of treatment modalities through a cost-effectiveness lens rather than curtailing specific treatments predicated strictly on economic considerations.

This study acknowledges some limitations that warrant consideration. First, our model predominantly draws upon data from clinical trials, which could introduce potential nuances and uncertainties into the findings. Notably, the long-term therapeutic benefits of combining utidelone with capecitabine for metastatic breast cancer are still under exploration. A more protracted follow-up may be beneficial in consolidating and updating the relevant data, which could provide a clearer understanding of this aspect. Second, our model does not fully encompass some aspects. Specifically, it does not factor in the costs associated with certain AEs (Zhang et al., 2011), particularly those of grade 3–4 with an incidence rate <5% and common grade 1–2 reactions such as peripheral neurotoxicity, hand-foot syndrome, and bone marrow suppression. Although these omissions may impart subtle variances to the study outcomes, our sensitivity analyses suggest that these factors, within the stipulated variability range, may not drastically sway the primary conclusions. Additionally, highlighting the role of utility values in pharmacoeconomic studies is essential. In the absence of quality-of-life data from the pertinent trials, our approach leaned on published utility values associated with metastatic breast cancer. Though our univariate sensitivity analyses hint at the influence of utility values for PFS and PD on the results, the insights from tornado diagrams provide a perspective that, even with adjustments within acceptable margins, ICERs would likely surpass the WTP threshold.

In conclusion, the therapeutic combination of utidelone and capecitabine is not presently an economically feasible alternative in chemotherapy for anthracycline- and taxane-refractory metastatic breast cancer. Nevertheless, in specific patient subgroups, the combination of utidelone with capecitabine may present a more cost-effective treatment choice than capecitabine monotherapy.

## Data Availability

The original contributions presented in the study are included in the article/supplementary material, further inquiries can be directed to the corresponding author.
